# High-intensity binge drinking is associated with cigarette smoking and e-cigarette use among US adults aged 40–64 years: Findings from the 2017 BRFSS survey

**DOI:** 10.18332/tid/122603

**Published:** 2020-06-15

**Authors:** Qian Wang

**Affiliations:** 1 School of Public Health, Shanghai Jiao Tong University School of Medicine, Shanghai, China

**Keywords:** high-intensity binge drinking, extreme high-intensity binge drinking, cigarette smoking, e-cigarette use, BRFSS

## Abstract

**INTRODUCTION:**

This study aims to assess the association of cigarette smoking, including e-cigarette use, with level of binge drinking, especially high-intensity and extreme high-intensity binge drinking, among a nationally representative sample of middle-aged US adults.

**METHODS:**

Data were derived from the 2017 Behavioral Risk Factor Surveillance System (BRFSS). The final sample consisted of 162748 respondents aged 40–64 years (48.7% male). Weighted distributions of sample characteristics were estimated by intensity of binge drinking. Pearson chi-squared tests were used to compare groups. Bivariate and multivariate logistic regressions were used to estimate crude and adjusted odds ratios to indicate the strength of the association between independent variables and each level of binge drinking.

**RESULTS:**

In all, 2.3% and 0.7% of the sample reported high-intensity and extreme high-intensity binge drinking, respectively. Past-month high-intensity and extreme high-intensity binge drinking were reported in 36.3% and 45.0% of smokers, respectively. Mental distress was positively associated with both levels of high-intensity binge drinking; having multiple chronic health conditions was negatively associated with past-month high-intensity binge drinking. Smokers had 3.27 (95% CI: 2.69–3.98) and 4.14 (95% CI: 3.12–5.49) times greater odds of reporting past-month high-intensity and extreme high-intensity binge drinking, respectively. E-cigarette users had 1.56 (95% CI: 1.01–2.42) times increased odds of reporting past-month high-intensity binge drinking, but not extreme high-intensity binge drinking. The largest odds were seen among dual users reporting extreme high-intensity binge drinking (AOR=6.05; 95% CI: 3.78–9.68) in the past month.

**CONCLUSIONS:**

Cigarette smoking and e-cigarette use were potentially strong risk factors for high-intensity binge drinking, with cigarette smoking associated with extreme high-intensity binge drinking.

## INTRODUCTION

Excessive alcohol consumption mainly refers to binge drinking (4 or more drinks for women, 5 or more drinks for men, within a 2-hour period) and heaving drinking (8 or more drinks a week for women, 15 or more drinks a week for men)^1^. Binge drinking differs from heavy drinking in that it typically raises the blood alcohol concentration (BAC) to 0.08 per cent or higher^1^. Binge drinking can place a heavy burden on society, and is considered as the most common, costly, and deadly pattern of excessive alcohol consumption, contributing to over half of the deaths and three-fourths of the economic costs incurred by excessive drinking^2,3^. However, evidence suggests that young people are increasingly consuming alcohol at two (8+/10+) or three (12+/15+) times the traditional (4+/5+) binge drinking threshold^4^, known as high-intensity binge drinking. For example, national surveys found 10.3–13.0% of those aged 18–26 years consumed 10+ drinks, and 4–5% consumed 15+ drinks, with 9.5 drinks as the average number of drinks consumed in one sitting^5,6^. In recent years, high-intensity binge drinking has not decreased in the same way that traditional binge drinking has^7^. Given the link between binge drinking and adverse outcomes, those who engage in high-intensity binge drinking may be at increased risk for acute health problems such as alcohol intoxication or poisoning, and long-term health problems such as alcohol use disorder^8^.

Worldwide, drinking and cigarette smoking are both leading risk factors for morbidity and premature mortality, and they commonly co-occur. Research within the adolescent and young adult population has consistently found drinking associated with the initiation and escalation of cigarette smoking^9^, while smokers may be at elevated risk of engaging in heavy drinking compared with non-smokers^10^. Heavier alcohol consumption combined with cigarette smoking is linked to a markedly greater risk of smoking-related diseases such as oral, pharyngeal, laryngeal, and esophageal cancers, and worsened brain morphology and functions^11,12^. A 30-year cohort study found men who smoked and consumed 15+ drinks per week had the highest all-cause mortality^13^. However, the majority of these studies measured alcohol use as the average number of drinks consumed per week or month, level of binge drinking was not directly assessed. A few studies examined the co-occurrence of binge drinking and smoking among the adolescent population, for example, Johnson et al.^14^ analyzed data from 4000 adolescents aged 13–18 years and found adolescent smokers likely to be binge drinkers (5+ drinks on the same occasion) and vice versa, highlighting the need for a dual focus in prevention and early intervention. In another study, Weitzman and Chen^15^ analyzed data from 10924 students across 120 colleges and found first-year students, especially female students, who picked up binge drinking (4+ for women and 5+ in a drinking occasion for men) in college had higher odds of smoking, again stressing the need to identify co-occurring smoking and binge drinking prevention and treatment models. However, research examining binge drinking and cigarette smoking has rarely extended to include high-intensity binge drinking, furthermore, such research is scarce among older populations.

According to the 2011–2017 Behavioral Risk Factor Surveillance System (BRFSS), the largest increase in the total number of binge drinks per US adult who reported binge drinking was observed among those aged 35–44 years (+26.7%) and 45–64 years (+23.1%), while a decrease was observed among those aged 18–24 years (-12.0%) and 25–34 years (-3.4%)^16^. Compared with adolescents and young adults, middle-aged adults often carry diverse responsibilities ranging from family-related to work-related domains, and binge drinking may place middle-aged adults at increased risk for adverse consequences or exacerbate age-related physiological changes. For example, an analysis of the 2010–2012 National Vital Statistics System revealed that an overwhelming 75.7% of alcohol-poisoning deaths involved adults aged 35–64 years^17^. A study found US adults aged ≥50 years and who binged more than 2 days per week had 64% greater odds of experiencing insomnia^18^. A longitudinal study assessing patterns of binge drinking (<6 versus ≥6 drinks per session) and progression of carotid atherosclerosis during a period of 11 years among Finnish middle-aged adults (mean age: 51.7±6.7 versus 49.7±6.8 years) found binge drinking of ≥6 drinks per session was significantly associated with increased atherosclerosis progression, even after controlling for demographic covariates and total alcohol consumption^19^. However, the majority of these studies on binge drinking by the middle-aged adult population have focused on the traditional binge drinking threshold, high-intensity binge drinking among this age group is understudied. Recent calls for research on high-intensity binge drinking suggest to examine its prevalence and associated characteristics within, as well as outside, the college environment^8^. Therefore, it is important to examine the phenomenon among the middle-aged adult population.

Compared with young (18–24 years) and older adults (≥65 years), middle-aged US adults also seem to have higher rates of cigarette smoking. According to the 2018 National Health Interview Survey (NHIS), 16.3% adults aged 44–64 years currently smoked cigarettes, while only 7.8% of those aged 18–24 years currently smoked cigarettes, and 8.4% of those aged ≥65 years currently smoked cigarettes^20^. Cigarette smoking was linked to poorer general intelligence, visuospatial learning, memory, and fine motor dexterity among middle-aged adults^21^, which may exacerbate aging-related changes in health. Electronic cigarette usage and correlates among adolescents and young adults have been extensively studied since its growth in popularity. However, recent statistics indicate a significant increase in current e-cigarette use among middle-aged adults, recreationally or as smoking cessation aids^22,23^. Yet, the health-related impact of e-cigarettes is not yet well-understood^24^, nor is its association with binge drinking, or high-intensity binge drinking, well-studied among middle-aged adults.

Therefore, we attempted to fill this gap in the literature through examining the link between levels of binge drinking, especially high-intensity binge drinking, and cigarette smoking and e-cigarette use among nationally representative US adults aged 40–64 years. The aim of the current study was to shed light on the prevalence of high-intensity binge drinking among middle-aged adults, and to examine its association with a key risk factor of morbidity and mortality – cigarette smoking in this population. Findings of this study will have important implications for treatment and intervention strategies.

## METHODS

### Study population

Data for this study were from the 2017 Behavioral Risk Factor Surveillance System (BRFSS). BRFSS is a telephone-based (combined landlines and mobile phones) survey that collects data among a nationally representative sample of non-institutionalized adults (≥18 years) in the US. It is developed and conducted annually by the CDC in conjunction with state health departments in all 50 states as well as the District of Columbia and US territories. The BRFSS utilizes a complex multistage cluster sampling design to adjust for non-response and selection bias, and a weighting method called iterative proportional fitting or raking was used to weight the data^25^. All 50 states as well as the District of Columbia, Guam, and Puerto Rico assessed alcohol consumption and use of tobacco products (including e-cigarettes) in 2017. For the purpose of the current investigation, we restricted our sample to middle-aged respondents aged 40–64 years that also had non-missing responses to items assessing levels of binge drinking. A total of 174196 respondents fell into this age group in the 2017 BRFSS.

### Measures

Level of binge drinking was assessed by two questions: ‘During the past 30 days, how many days per week or per month did you have at least one drink of any alcoholic beverage such as beer, wine, malt beverage, or liquor?’ and ‘During the past 30 days, what is the largest number of drinks you had on any occasion?’. Responses were coded to reflect level of binge drinking for men and women, respectively. Participants who reported no drinks during the past 30 days were coded as having 0 drinks. Light drinking was defined as 1–3 drinks for women and 1–4 drinks for men in one sitting. Traditional standard binge drinking was defined as 4+ drinks for women and 5+ drinks for men in one sitting1. High-intensity binge drinking was defined as 8+ drinks for women and 10+ drinks for men in one sitting^4^. Extreme high-intensity binge drinking was defined as 12+ drinks for women and 15+ drinks for men in one sitting^4^.

Current smoking and e-cigarette use were assessed by combining responses to two items: ‘adults who are current smokers (Yes or No)’ and ‘adults who are current e-cigarette users (not currently using e-cigarettes, current e-cigarette user)’. Responses to the two items were summed and coded to reflect four combinations: non-user, smoker only, e-cigarette user only, and dual user.

Mental distress was assessed by the item: ‘Now thinking about your mental health, which includes stress, depression, and problems with emotions, for how many days during the past 30 days was your mental health not good?’. We controlled for mental distress in the analysis because it was found to be associated with traditional standard binge drinking and smoking among adults aged ≥50 years^26,27^. Responses ranged from ‘none’ to ‘30 days’. To better present its estimated prevalence, we grouped responses into three categories: 0, 1–13 and 14–30 days/month.

Presence of chronic health conditions was also included in the analysis because existing research indicates that older adults (≥50 years) in good health were more likely to binge drink than those with multimorbidity^28^. It was assessed by asking respondents whether they were ever told by a doctor, nurse or other health professional that they had the following nine chronic health conditions: a heart attack (myocardial infarction), angina or chronic heart disease, a stroke, asthma, any type of cancer, chronic obstructive pulmonary disease (COPD) or emphysema or chronic bronchitis, arthritis, kidney disease, and diabetes. Answer options included ‘yes’ or ‘no’ for each of the conditions, and a new variable was created by summing responses across these nine conditions. We grouped the summed responses into 0, 1, 2, and 3–9 types.

We also controlled for demographic covariates that were commonly associated with binge drinking, which included age in five-year groups (40–44, 45–49, 50–54, 55–64, and 60–64 years), gender (male, female), race and ethnicity (non-Hispanic White, non-Hispanic Black, Hispanic, and Other), education (high school or below, attended college, college/technical school graduate), marital status (single, married/partnered, divorced/widowed/separated), and employment status (unemployed, employed, inactive). For prevalence estimates, age was analyzed in its original five-year groups; in the subsequent logistic regression analysis, age was analyzed as a continuous variable. We coded those that reported ‘Out of work for 1 year or more’ or ‘Out of work for less than 1 year’ as unemployed, those reporting ‘employed for wages’ or ‘self-employed’ as employed, and those reporting to be ‘a homemaker’, ‘a student’, ‘retired’, or ‘unable to work’ as inactive.

### Statistical analysis

All statistical analyses were performed using SAS Version 9.4 (SAS Institute Inc., Cary, North Carolina, USA). A total of 174196 respondents aged 40–64 years with non-missing responses to level of binge drinking were included initially. Percentage of non-eligible responses (‘don't know’, ‘refused’, or ‘missing’) was 0.20% for education, 1.48% for race and ethnicity, 0.36% for marital status, 0.63% for employment status, 1.23% for mental distress, 3.55% for chronic health conditions, and 0.01% for smoking and e-cigarette use. With non-eligible responses excluded from analysis, the final sample consisted of 162748 unique cases. Because the BRFSS employs complex survey designs, SAS survey procedures (e.g. SURVEYFREQ and SURVEYLOGISTIC) were used to account for weighting, clustering, and stratification of the sample. Weighted distributions of sample characteristics were calculated for the total sample as well as by level of binge drinking intensity. Pearson’s chi-squared test was used to determine if there was a difference between two or more groups of categorical independent variables. Crude and adjusted odds ratios and their corresponding 95% confidence intervals (CIs) were calculated to indicate the strength of the association between independent variables and level of binge drinking intensity.

## RESULTS

### Sample characteristics

Table 1 presents weighted distributions of sample characteristics by level of binge drinking intensity. The sample was made up of 48.7% males and 51.3% females, 62.5% were aged ≥50 years, 31.0% graduated from college or technical school, 68.4% were either ‘employed for wages’ or ‘self-employed’, and 66.8% were ‘married’ or ‘a member of an unmarried couple’ (partnered). During the past 30 days, 11.4% reported traditional standard binge drinking, 2.3% reported high-intensity binge drinking, and 0.7% reported extreme high-intensity binge drinking. The prevalence of smokers was 21.6% among those reporting traditional standard binge drinking, 36.3% and 45.0% among those reporting high-intensity and extreme high-intensity binge drinking, respectively.

**Table 1:** Sample characteristics by past-month level of binge drinking intensity among US adults aged 40–64 years, BRFSS 2017 (N=162748)

*Characteristics*	*Total*	*Past-month level of binge drinking intensity ^a^*					*p*
		*None*	*Light drinking*	*Traditional standard*	*High-intensity*	*Extreme high-intensity*	

	*n (%) ^b^*	*n (%)*	*n (%)*	*n (%)*	*n (%)*	*n (%)*	
**Total**	162748 (100.0)	73637 (45.5)	67112 (40.1)	17713 (11.4)	3154 (2.3)	1132 (0.7)	
**Age** (years)							
40–44	21622 (19.2)	9034 (17.9)	8585 (18.9)	3029 (22.5)	692 (29.7)	282 (30.1)	<0.001
45–49	26135 (18.2)	10919 (17.2)	10836 (18.3)	3363 (20.5)	719 (24.9)	298 (25.5)	
50–54	32166 (21.5)	14259 (21.3)	13127 (21.2)	3843 (23.3)	690 (23.1)	247 (23.9)	
55–64	39161 (20.4)	18119 (21.2)	16216 (20.5)	4014 (18.9)	632 (14.2)	180 (10.4)	
60–64	43664 (20.6)	21306 (22.5)	18348 (21.1)	3464 (14.7)	421 (8.2)	125 (10.1)	
**Gender**							
Male	72520 (48.7)	28941 (43.1)	30327 (49.2)	9951 (60.6)	2426 (80.8)	875 (77.3)	<0.001
Female	90228 (51.3)	44696 (56.9)	36785 (50.8)	7762 (39.4)	728 (19.2)	257 (22.7)	
**Education**							
High school or below	51611 (38.9)	29677 (48.5)	14638 (27.7)	5319 (35.9)	1376 (52.3)	601 (60.9)	<0.001
Attended college	44790 (30.1)	20911 (29.0)	17787 (31.3)	4863 (31.1)	923 (28.5)	306 (28.1)	
College/technical school graduate	66347 (31.0)	23049 (22.5)	34687 (41.0)	7531 (33.0)	855 (19.2)	225 (11.0)	
**Race/ethnicity**							
Non-Hispanic White	125400 (66.0)	52340 (59.0)	55199 (71.6)	14530 (72.5)	2498 (72.3)	833 (71.0)	<0.001
Non-Hispanic Black	13609 (12.0)	7644 (13.8)	4685 (10.8)	1057 (9.4)	160 (8.8)	63 (6.0)	
Hispanic	12718 (14.9)	7316 (18.5)	3873 (10.9)	1186 (13.9)	247 (15.7)	96 (16.0)	
Non-Hispanic Other	11021 (7.2)	6337 (8.6)	3355 (6.6)	940 (4.3)	249 (3.3)	140 (7.0)	
**Marital status**							
Single	17877 (10.8)	9675 (12.6)	5723 (8.6)	1852 (10.2)	443 (14.3)	184 (16.9)	<0.001
Divorced/widowed/separated	40031 (22.4)	20503 (25.1)	13902 (18.9)	4339 (22.5)	880 (26.5)	407 (33.8)	
Married/partnered	104840 (66.8)	43459 (62.3)	47487 (72.5)	11522 (67.3)	1831 (59.2)	541 (49.3)	
**Employment status**							
Unemployed	8344 (5.8)	4489 (6.9)	2682 (4.6)	912 (5.4)	181 (5.9)	80 (9.5)	<0.001
Inactive	43848 (25.8)	26437 (34.6)	13609 (19.1)	3074 (16.7)	504 (15.5)	224 (17.7)	
Employed	110556 (68.4)	42711 (58.5)	50821 (76.3)	13727 (77.9)	2469 (78.5)	828 (72.8)	
**Mental distress** (days/month)							
0	106805 (66.1)	47081 (65.3)	45824 (68.2)	11281 (64.3)	1969 (59.1)	650 (53.5)	<0.001
1–13	35742 (21.5)	14897 (19.3)	15459 (23.0)	4441 (24.4)	711 (24.8)	234 (20.7)	
14–30	20201 (12.4)	11659 (15.4)	5829 (8.8)	1991 (11.4)	474 (16.1)	248 (25.8)	
**Chronic health conditions**							
0	79558 (51.2)	30978 (45.3)	36368 (55.3)	9891 (58.9)	1766 (59.5)	555 (51.0)	<0.001
1	47532 (28.6)	21272 (28.4)	19896 (29.5)	5184 (26.9)	848 (25.5)	332 (27.5)	
2	20563 (11.7)	11180 (13.8)	7179 (10.2)	1732 (9.5)	345 (8.7)	127 (10.5)	
3–9 types	15095 (8.5)	10207 (12.5)	3669 (5.1)	906 (4.7)	195 (6.3)	118 (11.0)	
**Current smoking e-cigarette use**							
Non-user	132922 (81.0)	59183 (80.6)	58184 (85.5)	13156 (73.8)	1877 (57.0)	522 (44.9)	<0.001
Dual user	2926 (2.0)	1435 (2.1)	868 (1.4)	443 (2.8)	114 (5.0)	66 (8.2)	
Smoker only	24917 (15.6)	12091 (16.0)	7366 (11.8)	3829 (21.6)	1109 (36.3)	522 (45.0)	
E-cigarette user only	1983 (1.3)	928 (1.3)	694 (1.2)	285 (1.7)	54 (1.7)	22 (1.9)	

a Light drinking: 1–3 drinks for females and 1–4 drinks for males.

Traditional standard binge drinking: 4+ drinks for females and 5+ drinks for males. High-intensity binge drinking: 8+ drinks for females and 10+ drinks for males. Extreme high-intensity binge drinking: 12+ drinks for females and 15+ drinks for males.

b All percentages are weighted.

Compared with lower drinking levels, prevalence of high-intensity binge drinking was higher among those who were 40–44 years of age (29.7%), male (80.8%), high school graduates or below (52.3%), single (14.3%) or divorced/widowed/separated (26.5%), employed (78.5%), mentally distressed for 1–13 days (24.8%) or 14–30 days (16.1%) during the past 30 days, without chronic health conditions (59.5%), smokers only (36.3%) or dual users (5.0%).

Compared with lower drinking levels, excluding high-intensity binge drinking, prevalence of extreme high-intensity binge drinking was higher among those who were 40–44 years of age (30.1%), male (77.3%), high school graduates or below (60.9%), single (16.9%) or divorced/widowed/separated (33.8%), currently unemployed (9.5%), mentally distressed for 14 days or more (25.8%) during the past 30 days, e-cigarette users (1.9%), smokers (45.0%) or dual and users (8.2%).

### Logistic regressions

Tables 2 and 3 summarize results from the bivariate and multivariate logistic regression models, respectively. In the bivariate logistic regression model, the crude odds of response were calculated for each explanatory variable separately. In the multivariate logistic regression model, the odds of response were calculated by adjusting for potential confounding factors.

In the bivariate analysis, smoking alone as well as smoking with concurrent e-cigarette use was associated with decreased odds of light drinking, but these odds were no longer significant in the multivariate analysis. E-cigarette use was associated with higher odds of all three levels of binge drinking in the bivariate analysis, but its association with extreme high-intensity binge drinking was no longer significant in the multivariate analysis.

After adjusting for all confounding factors, smokers had a higher likelihood of reporting binge drinking (AOR=1.94; 95% CI: 1.76–2.14), high-intensity binge drinking (AOR=3.27; 95% CI: 2.69–3.98) and extreme high-intensity binge drinking (AOR=4.14; 95% CI: 3.12–5.49). The largest odds of past-month high-intensity (AOR=3.79; 95% CI: 2.36–6.09) and extreme high-intensity binge drinking (AOR=6.05; 95% CI: 3.78–9.68) were seen among dual tobacco and e-cigarette users. However, neither smoking nor dual use was associated with past-month light drinking. Compared with smokers, the odds of past-month high-intensity binge drinking were smaller for e-cigarette only users (AOR=1.56; 95% CI: 1.01–2.42).

**Table 2:** Crude odds ratios (ORs) and corresponding 95% confidence intervals (CIs) for each explanatory variable of past-month level of binge drinking intensity among those aged 40–64 years, BRFSS 2017 (N=162748)

*Characteristics*	*Past-month level of binge drinking intensity ^a^*			
	*Light drinking*	*Traditional standard*	*High-intensity*	*Extreme high-intensity*

	*OR (95% CI)*	*OR (95% CI)*	*OR (95% CI)*	*OR (95% CI)*
**Age**	0.97 (0.95–0.98)***	0.86 (0.84–0.88)***	0.71 (0.67–0.75)***	0.70 (0.64–0.77)***
**Gender**				
Male	1.28 (1.23–1.34)***	2.04 (1.90–2.19)***	5.57 (4.57–6.77)***	4.50 (3.37–6.00)***
Female	1.00	1.00	1.00	1.00
**Education**				
High school or below	0.53 (0.50–0.56)***	0.69 (0.63–0.76)***	1.10 (0.91–1.32)	1.30 (1.00–1.69)***
Attended college	1.00	1.00	1.00	1.00
College/technical school graduate	1.70 (1.60–1.79)***	1.37 (1.26–1.49)***	0.87 (0.72–1.05)	0.51 (0.38–0.68)***
**Race/ethnicity**				
Non-Hispanic White	1.00	1.00	1.00	1.00
Non-Hispanic Black	0.65 (0.60–0.70)**	0.55 (0.49–0.63)**	0.52 (0.39–0.69)**	0.36 (0.25–0.54)***
Hispanic	0.49 (0.45–0.53)***	0.61 (0.54–0.69)**	0.69 (0.54–0.89)**	0.72 (0.48–1.07)
Non-Hispanic Other	0.63 (0.56–0.71)**	0.40 (0.33–0.49)***	0.32 (0.24–0.41)***	0.67 (0.41–1.10)
**Marital status**				
Single	0.59 (0.54–0.64)***	0.75 (0.67–0.84)***	1.19 (0.96–1.47)	1.69 (1.21–2.37)*
Divorced/widowed/separated	0.65 (0.61–0.68)***	0.83 (0.77–0.90)*	1.11 (0.93–1.33)	1.70 (1.32–2.19)*
Married/partnered	1.00	1.00	1.00	1.00
**Employment status**				
Unemployed	0.51 (0.46–0.56)***	0.58 (0.51–0.67)*	0.64 (0.48–0.85)*	1.10 (0.66–1.86)
Inactive	0.42 (0.40–0.45)***	0.36 (0.33–0.40)***	0.33 (0.27–0.42)***	0.41 (0.31–0.55)***
Employed	1.00	1.00	1.00	1.00
**Mental distress** (days/month)				
0	1.00	1.00	1.00	1.00
1–13	1.14 (1.08–1.21)***	1.28 (1.18–1.39)***	1.42 (1.14–1.77)*	1.31 (0.98–1.76)
14–30	0.55 (0.51–0.59)***	0.75 (0.68–0.84)***	1.16 (0.93–1.43)	2.05 (1.54–2.74)***
**Chronic health conditions**				
0	1.00	1.00	1.00	1.00
1	0.85 (0.81–0.90)***	0.73 (0.67–0.79)***	0.68 (0.58–0.81)*	0.86 (0.66–1.13)
2	0.60 (0.56–0.65)*	0.53 (0.48–0.59)*	0.48 (0.38–0.60)*	0.68 (0.49–1.02)
3–9 types	0.33 (0.31–0.36)***	0.29 (0.25–0.33)***	0.39 (0.28–0.54)***	0.78 (0.51–1.20)
**Current smoking e-cigarette use**				
Non-user	1.00	1.00	1.00	1.00
Dual user	0.64 (0.54–0.76)**	1.47 (1.18–1.82)*	3.35 (2.16–5.21)**	6.95 (4.43–10.92)***
Smoker only	0.70 (0.65–0.75)**	1.48 (1.35–1.61)*	3.22 (2.69–3.84)***	5.05 (3.96–6.45)***
E-cigarette user only	0.86 (0.71–1.04)	1.39 (1.10–1.77)*	1.79 (1.17–2.74)*	2.61 (1.20–5.68)**

a Light drinking: 1–3 drinks for females and 1–4 drinks for males.

Traditional standard binge drinking: 4+ drinks for females and 5+ drinks for males. High-intensity binge drinking: 8+ drinks for females and 10+ drinks for males. Extreme high-intensity binge drinking: 12+ drinks for females and 15+ drinks for males.

* p<0.05.

** p<0.01.

*** p<0.001.

**Table 3:** Adjusted odds ratios (AOR) and corresponding 95% confidence intervals (CIs) for each explanatory variable of past-month level of binge drinking intensity among those aged 40–64 years, BRFSS 2017 (N=162748)

*Characteristics*	*Past-month level of binge drinking intensity ^a^*			
	*Light drinking*	*Traditional standard*	*High-intensity*	*Extreme high-intensity*

	*AOR (95% CI)*	*AOR (95% CI)*	*AOR (95% CI)*	*AOR (95% CI)*
**Age**	1.03 (1.01–1.05)**	0.92 (0.90–0.95)***	0.76 (0.72–0.80)***	0.74 (0.67–0.82)***
**Gender**				
Male	1.27 (1.21–1.33)***	1.98 (1.84–2.13)***	5.26 (4.33–6.38)***	4.26 (3.18–5.72)***
Female	1.00	1.00	1.00	1.00
**Education**				
High school or below	0.59 (0.55–0.63)***	0.69 (0.62–0.75)***	0.95 (0.79–1.13)	1.09 (0.82–1.43)
Attended college	1.00	1.00	1.00	1.00
College/technical school graduate	1.52 (1.43–1.61)***	1.33 (1.22–1.46)***	0.95 (0.78–1.15)	0.62 (0.46–0.84)***
**Race/ethnicity**				
Non-Hispanic White	1.00	1.00	1.00	1.00
Non-Hispanic Black	0.79 (0.73–0.86)**	0.62 (0.54–0.71)**	0.49 (0.36–0.67)***	0.31 (0.21–0.47)***
Hispanic	0.61 (0.56–0.66)***	0.69 (0.60–0.79)**	0.64 (0.50–0.84)*	0.69 (0.45–1.06)
Non-Hispanic Other	0.52 (0.46–0.60)***	0.34 (0.28–0.41)***	0.28 (0.22–0.37)***	0.67 (0.41–1.10)
**Marital status**				
Single	0.70 (0.64–0.76)***	0.78 (0.70–0.88)***	1.00 (0.78–1.27)	1.32 (0.90–1.93)
Divorced/widowed/separated	0.83 (0.78–0.88)*	1.00 (0.91–1.09)	1.20 (0.98–1.46)	1.54 (1.18–2.02)*
Married/partnered	1.00	1.00	1.00	1.00
**Employment status**				
Unemployed	0.69 (0.62–0.77)**	0.68 (0.59–0.79)**	0.56 (0.41–0.76)**	0.72 (0.40–1.30)
Inactive	0.58 (0.55–0.62)***	0.53 (0.48–0.58)***	0.50 (0.39–0.64)***	0.41 (0.30–0.57)***
Employed	1.00	1.00	1.00	1.00
**Mental distress** (days/month)				
0	1.00	1.00	1.00	1.00
1–13	1.23 (1.16–1.31)***	1.44 (1.32–1.56)***	1.67 (1.35–2.07)**	1.41 (1.05–1.89)*
14–30	0.88 (0.81–0.95)***	1.14 (1.01–1.29)*	1.56 (1.22–2.00)**	2.14 (1.58–2.91)***
**Chronic health conditions**				
0	1.00	1.00	1.00	1.00
1	0.94 (0.89–1.00)	0.81 (0.74–0.88)***	0.78 (0.65–0.94)**	0.92 (0.69–1.22)
2	0.76 (0.71–0.82)*	0.65 (0.58–0.73)*	0.58 (0.45–0.75)*	0.71 (0.49–1.03)
3–9 types	0.51 (0.46–0.56)***	0.39 (0.34–0.46)***	0.50 (0.34–0.72)**	0.82 (0.53–1.27)
**Current smoking e-cigarette use**				
Non-user	1.00	1.00	1.00	1.00
Dual user	0.95 (0.79–1.15)	2.04 (1.60–2.59)*	3.79 (2.36–6.09)**	6.05 (3.78–9.68)***
Smoker only	1.03 (0.96–1.11)	1.94 (1.76–2.14)***	3.27 (2.69–3.98)***	4.14 (3.12–5.49)**
E-cigarette user only	1.03 (0.85–1.25)	1.51 (1.18–1.92)*	1.56 (1.01–2.42)*	1.95 (0.88–4.34)

a Light drinking: 1–3 drinks for females and 1–4 drinks for males.

Traditional standard binge drinking: 4+ drinks for females and 5+ drinks for males. High-intensity binge drinking: 8+ drinks for females and 10+ drinks for males. Extreme high-intensity binge drinking: 12+ drinks for females and 15+ drinks for males.

* p<0.05.

** p<0.01.

*** p<0.001.

In the bivariate analysis, moderate level of mental distress (1–13 days/month) was associated with increased odds of binge drinking and high-intensity binge drinking, but it was associated with increased odds of extreme high-intensity binge drinking only in the multivariate analysis. Higher level of mental distress (≥14 days/month) was associated with lower odds of binge drinking in the bivariate analysis, but the association became positive in the multivariate analysis, suggesting underlying interaction effects with one or more variables. Those with higher level of mental distress (≥14 days/month) during the past month had twice (AOR=2.14; 95% CI: 1.58–2.91) increased odds of reporting extreme high-intensity binge drinking.

Having at least 1 chronic health condition was associated with lower odds of standard binge drinking and high-intensity binge drinking in the bivariate as well as multivariate analyses. Those with 3–9 types of chronic health conditions were 61% (95% CI: 0.34–0.46) and 50% (95% CI: 0.34–0.72) less likely to report traditional standard binge drinking and high-intensity binge drinking, respectively.

Age was associated with lower odds of all three levels of binge drinking in both bivariate and multivariate analyses; the odds of traditional standard binge drinking, high-intensity binge drinking, and extreme high-intensity binge drinking decreased by 8% (95% CI: 0.90–0.95), 24% (95% CI: 0.72–0.80), and 26% (95% CI: 0.67–0.82), respectively, as age increased. Compared with females, males had 1.98 times (95% CI: 1.84–2.13), 5.26 times (95% CI: 4.33–6.38) and 4.26 times (95% CI: 3.18–5.72) greater odds of reporting high-intensity and extreme high-intensity binge drinking, respectively. Compared with non-Hispanic Whites, all other races/ethnicities had lower odds of reporting binge drinking and high-intensity binge drinking, with the largest decreased odds seen among those of non-Hispanic other race. Non-Hispanic Blacks also reported 69% (95% CI: 0.21–0.47) lower odds of reporting extreme-high intensity binge drinking. Being single or divorced/widowed/separated was associated with lower odds of binge drinking but higher odds of extreme high-intensity binge drinking in the bivariate analysis. However, the association between being single and reporting extreme high-intensity binge drinking was no longer statistically significant in the multivariate analysis; only being divorced/widowed/separated had 1.54 (95% CI: 1.18–2.02) times increased odds of reporting extreme high-intensity binge drinking.

## DISCUSSION

To our knowledge, the current investigation is the first to examine prevalence of high-intensity and extreme high-intensity binge drinking and its relation with cigarette smoking and e-cigarette use among a nationally representative sample of middle-aged US adults. We found the percentage of smokers increased with an increment in levels of binge drinking, while 21.6% of those reporting traditional standard binge drinking were smoking, this percentage increased to 36.3% among those reporting high-intensity binge drinking, and to 45.0% among those reporting extreme high-intensity binge drinking, higher than the percentage of smokers among non-drinkers (16.0%) and light drinkers (11.8%). Furthermore, after adjusting for confounding factors, those who were smoking and co-using e-cigarettes had significantly larger odds of reporting all three levels of binge drinking but not light drinking, with the largest odds observed among dual users reporting extreme high-intensity binge drinking. Meanwhile, e-cigarette only users had relatively smaller increased odds of reporting traditional standard binge drinking and high-intensity binge drinking, but not extreme high-intensity binge drinking. Our main finding implies that cigarette smoking rather than e-cigarette use may be a stronger risk factor for high and extremely high-intensity binge drinking for middle-aged adults.

Alcohol consumption often co-occurs with cigarette smoking, as their association has long been documented in both clinical and non-clinical samples^29^. An estimated 80% to 95% of alcoholics were smokers, while smokers had 4 to 10 times increased odds of developing alcohol use disorders^30^. The close association between drinking and smoking has meaningful implications for treatment and intervention. Some studies found alcohol use or binge drinking decreased following smoke cessation for most smokers, however, those who were consuming large amounts of alcohol prior to smoke cessation were most likely to continue heavy alcohol use even after smoke cessation^31,32^. Some studies found standard binge drinking could impede smoking cessation. Kahler et al.^33^ reported that compared with moderate non-binge drinking, standard binge drinking doubled smoking lapse. In another study, Kahler et al.^33^ analyzed data on smokers from four countries and found those who binge drank had low rates of quitting smoking, furthermore, sustained smoking cessation did not lead to significant reductions in drinking, suggesting that smoking cessation interventions alone were unlikely to affect hazardous drinking^34^. However, findings on concurrent treatment have been inconclusive. For example, Joseph et al.^35^ found concurrent smoking cessation intervention did not benefit intensive treatment for alcohol dependence or abuse. A broad assessment of the effects of concurrent treatment of drinking and smoking may be out of scope of the current investigation, nonetheless, our finding that smoking was significantly correlated with higher levels of binge drinking among middle-aged adults may propel the need to call for future research examining their synergistic effects and the corresponding influence on treatment or intervention outcomes.

A few studies examined e-cigarette use in relation to standard binge drinking among US adolescents and young adults. For example, Hefner et al.^36^ found binge consuming 5 drinks or more was associated with increased odds of ever tried e-cigarettes among 631 students at a northeastern University. Littlefield et al.^37^ found odds of standard binge drinking increased among traditional cigarette users, e-cigarette users, and dual users at a state college. Yet, research assessing binge drinking in relation to e-cigarette use among the adult population is scarce, though evidence suggests that e-cigarette use may be a risk factor of problematic drinking^38^. Our finding that e-cigarette use alone was positively associated with binge drinking and high-intensity binge drinking among middle-aged adults extends similar findings in the younger population to the middle-aged population. Our finding further indicates that e-cigarette use alone could be a strong risk factor of higher levels of binge drinking among middle-aged adults, thus, use of e-cigarettes should be included in future studies on binge drinking to improve our understanding of its impact. Further research is also needed to clarify how e-cigarette use could add to the effect of smoking on levels of binge drinking, as we found dual users had the largest odds of reporting all levels of binge drinking than users of either substance alone.

The association between alcohol use and certain mental disorders such as depression has been more extensively studied than its association with mental distress per se. Although a positive association between mental distress and binge drinking has been tentatively documented among the older population^39^, the definition and assessment of mental distress varied, and thus warrants further validation. In regard to the item in the 2017 BRFSS that assesses mental distress and describes symptoms in broad terms that include stress, depression, and problems with emotions, our finding confirms its association with binge drinking and extends this association to higher levels of binge drinking among the middle-aged population, as the largest odds were seen among those having mental distress for 14 days or more during the past month and reporting extreme high-intensity binge drinking. Experiencing mental distress may prompt people to seek relief in alcohol consumption, but heavy alcohol consumption may also lead to mental distress and risks of suicide^40^. Further studies are needed to confirm the temporal sequence of binge drinking and mental distress measured using similar items. Heavy alcohol consumption is the major cause for a variety of chronic diseases, and a component cause for more than 200 other diseases conditions^41^. Although we were not able to derive causality from cross-sectional data, our finding demonstrated that binge drinking was less likely to occur among people with multiple chronic health conditions, which is consistent with existing research^42^, perhaps because some people may decide to cease binge drinking once they develop alcohol-related chronic health conditions^42^.

### Limitations

The current study has several limitations. First, the self-report nature of the data may lead to social desirability bias, where participants answer in a more socially acceptable way. Second, the cross-sectional nature of the data limits our ability to derive any direction of causality between smoking and high levels of binge drinking. Further longitudinal research is needed to clarify such directions. Nonetheless, the representativeness of the BRFSS data allows us to examine the link between smoking and high levels of binge drinking in the general middle-aged US adult population, so as to potentially strengthen the generalizability of our findings.

## CONCLUSIONS

The findings of the current study suggest that cigarette smoking could be a strong risk factor for high-intensity and extreme high-intensity binge drinking among middle-aged US adults. Further research is needed to confirm our finding that e-cigarette use alone could be an independent risk factor for binge drinking and high-intensity binge drinking, and to examine how dual use can add to the impact of cigarette smoking on levels of binge drinking.
